# Impact of current smoking on 2-year clinical outcomes between durable-polymer-coated stents and biodegradable-polymer-coated stents in acute myocardial infarction after successful percutaneous coronary intervention: Data from the KAMIR

**DOI:** 10.1371/journal.pone.0205046

**Published:** 2018-10-05

**Authors:** Yong Hoon Kim, Ae-Young Her, Myung Ho Jeong, Byeong-Keuk Kim, Sung-Jin Hong, Dong-Ho Shin, Jung-Sun Kim, Young-Guk Ko, Donghoon Choi, Myeong-Ki Hong, Yangsoo Jang

**Affiliations:** 1 Division of Cardiology, Department of Internal Medicine, Kangwon National University School of Medicine, Chuncheon, South Korea; 2 Department of Cardiology, Cardiovascular Center, Chonnam National University Hospital, Gwangju, South Korea; 3 Division of Cardiology, Severance Cardiovascular Hospital, Yonsei University College of Medicine, Seoul, South Korea; Azienda Ospedaliero Universitaria Careggi, ITALY

## Abstract

**Objective:**

Data concerning the effect of current smoking on solely new-generation drug-eluting stents (DES) are limited. We investigated the impact of current smoking on 2-year clinical outcomes between durable-polymer (DP)-coated DES (zotarolimus-eluting [ZES] and everolimus eluting [EES]) and biodegradable-polymer (BP)-coated biolimus-eluting stent (BES) in acute myocardial infarction (AMI) patients after successful percutaneous coronary intervention (PCI).

**Methods:**

Finally, a total of 8357 AMI patients with current smoking underwent successful PCI with new-generation DES (ZES, EES, and BES) were enrolled and divided into three groups as ZES (n = 3199), EES (n = 3987), and BES group (n = 1171). The primary endpoint was the occurrence of major adverse cardiac events (MACE) defined as all-cause death (cardiac death [CD] or non-cardiac death), recurrent AMI (re-MI), any revascularization (target lesion revascularization [TLR], target vessel revascularization [TVR], and non-TVR). The secondary endpoint was the incidence of definite or probable stent thrombosis (ST).

**Results:**

The 2-year adjusted hazard ratio (HR) of MACE for ZES vs. EES (1.055; 95% confidence interval [CI], 0.843–1.321; p = 0.638), ZES vs. BES (HR, 0.885; 95% CI, 0.626–1.251; p = 0.488), EES vs. BES (HR, 0.889; 95% CI, 0.633–1.250; p = 0.499), and ZES/EES vs. BES (HR, 0.891; 95% CI, 0.648–1.126; p = 0.480) were similar. The occurrence of ST after adjustment were also comparable. In addition, the 2-year adjusted HR for all-cause death, CD, re-MI, TLR, TVR, and non-TVR were not different.

**Conclusions:**

In this study, DP-DES and BP-DES showed comparable safety and efficacy during 2-year follow-up periods. Therefore, DP-DES or BP-DES are equally acceptable in AMI patients with current smoking undergoing PCI.

## Introduction

Cigarette smoking is one of important risks of coronary artery disease, including stable angina, acute coronary syndrome (ACS), and sudden cardiac death and other diverse vascular disease including stroke, aortic aneurysm, and peripheral vascular disease. [1–3] The relationship between smoking and myocardial infarction (MI) is well known. [4, 5] The main contributable mechanisms of cigarette smoking on increased mortality and morbidity of cardiovascular disease are related to oxidative stress, increased thrombin generation, platelet aggregation, inflammation, and endothelial dysfunction. [6] Some postulated suggestions are that nicotine may play an important role in atherogenesis and involved in enhanced endothelial cell proliferation and migration, and accelerate intimal hyperplasia in vitro and animal study. [7, 8] Persistent long-term cigarette smoking may cause luminal narrowing of the coronary arteries, arterioles, and microvasculature. [9] Although AMI milieu tends to higher thrombotic condition compared to stable coronary artery disease, drug-eluting stents (DES) implantation during primary percutaneous coronary intervention (PCI) or staged PCI were commonly done from the beginning of DESs era up to now. Compare to bare-metal stents (BMS), DES have reduced target lesion revascularization (TLR) by inhibition of neointimal hyperplasia but increased risk of fatal stent thrombosis (ST) is one of major concerns. [10, 11] To overcome these limitations, stent platforms and polymers have rapidly evolved during a short period. Newer anti-proliferative drugs and more biocompatible polymers have been adapted in reducing the rate of late ST. [12] At present, second-generation (2G)-DES have nearly replaced first-generation (1G)-DES during PCI in our routine daily clinical practice. However, there are limited studies concerning the effect of current smoking on solely new-generation DES, especially the milieu of AMI. [13, 14] The aim of this study was to investigate the impact of current smoking on 2-year clinical outcomes between durable-polymer (DP)-coated stents (zotarolimus-eluting [ZES] and everolimus eluting [EES]) and biodegradable-polymer (BP)-coated biolimus-eluting stent (BES) in AMI patients after successful PCI

## Materials and methods

### Study population

In this study, the data were obtained from the Korea AMI Registry (KAMIR). KAMIR is a nationwide, prospective, observational on-line registry in South Korea since November 2005 to evaluate current epidemiology, short-term and long-term clinical outcomes of patients with AMI. Fifty-three high-volume University or community hospitals with facilities for primary PCI and onsite cardiac surgery participated in this study. These data collected by a trained study coordinator using a standardized web-based case report form at each site of South Korea. Details of the registry can be found at the KAMIR website (http://www.kamir.or.kr).

**Fig 1:**
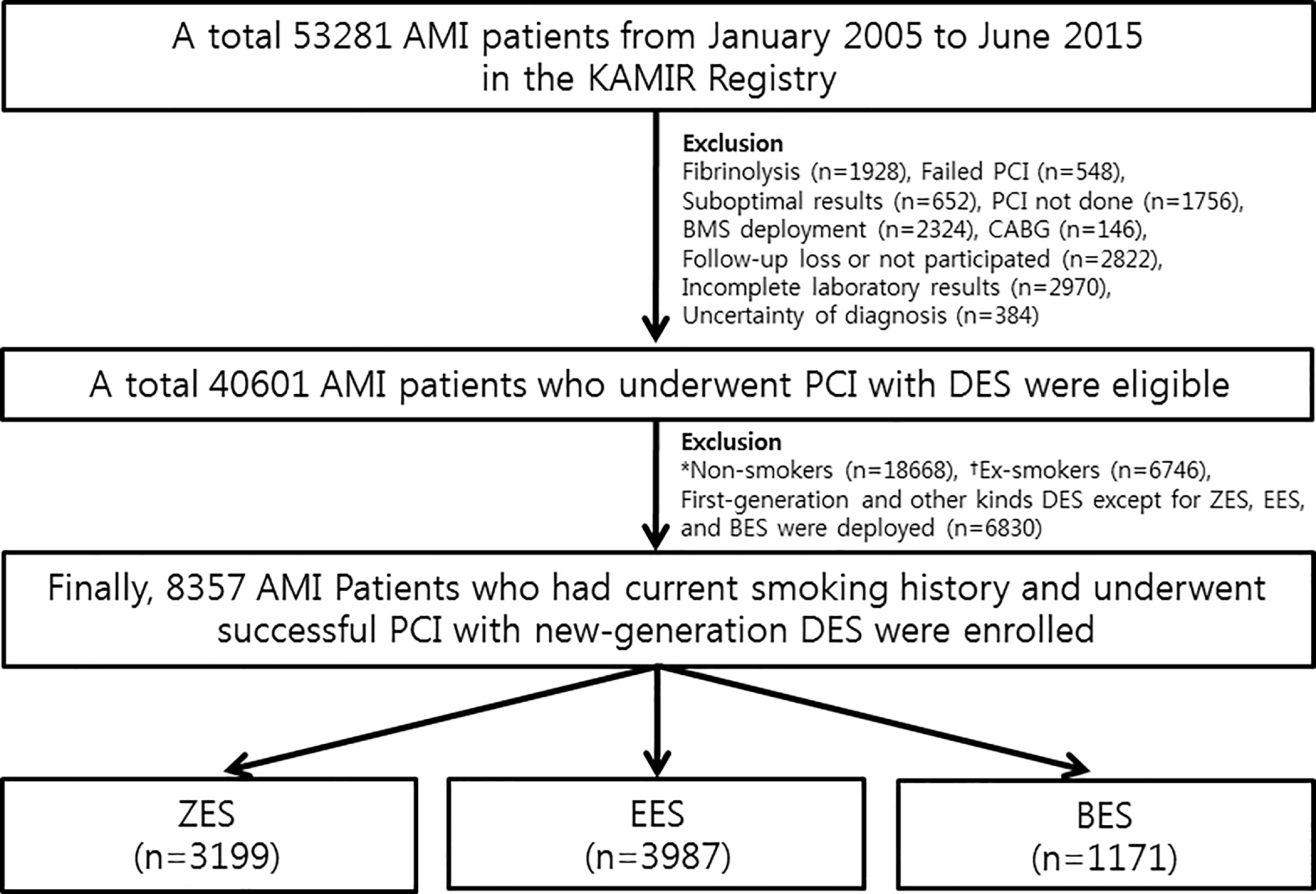
Flow chart. AMI, acute myocardial infarction; KAMIR, Korea Acute Myocardial Infarction Registry; PCI, percutaneous coronary intervention; BMS, bare-metal stent; CABG, coronary artery bypass graft; DES, drug-eluting stents; ZES, zotarolimus-eluting stents; EES, everolimus-eluting stents; BES, biolimus-eluting stents. *Non-smoker was defined as who did not regularly smoke at any time. ^†^Ex-smoker was defined as who had stopped smoking for more than 1 year before the index PCI

This study was a non-randomized, multicenter, observational, retrospective study. A total 53281 AMI patients between January 2005 and June 2015 in the KAMIR Registry were evaluated. Among them, the patients who had these conditions were excluded: (1) fibrinolysis was done (n = 1982, 3.7%), (2) failed PCI (n = 548, 1.0%), (3) suboptimal results (n = 652, n = 1.2%), (4) PCI was not done (n = 1756, 3.3%), (5) bare-metal stent (BMS) deployment (n = 2324, 4.4%) (6) CABG was done (n = 146, 0.3%), (7) follow-up loss or not participated (n = 2822, 5.3%), (8) incomplete laboratory results (n = 2970, 5.6%), (9) uncertainty of diagnosis (n = 384, 0.7%), (10) non-smokers (n = 18668, 35.0%), (11) Ex-smokers (n = 6746, 12.7%), (12) 1G or other kinds of DES except for ZES, EES, and BES (n = 6830, 12.8%). Finally, a total of 8357 AMI patients underwent successful PCI with new-generation DES (ZES, EES, and BES) were enrolled and divided into three groups as ZES (Resolute Integrity stent; Medtronic, Inc., Minneapolis, MN, n = 3199, 38.3%), EES (Xience Prime stent, Abbott Vascular, Santa Clara, CA; or Promus Element stent, Boston Scientific, Natick, MA, n = 3987, 47.7%), and BES group (BioMatrix Flex stent, Biosensors International, Morges, Switzerland; or Nobori stent, Terumo Corporation, Tokyo, Japan, n = 1171, 14.0%) (Fig 1). The ZES and EES group are belong to DP-DES and the BES group is belong to BP-DES. This study protocol was approved by the ethics committee at each participating center and the Chonnam National University Hospital Institutional Review Board (IRB) ethics committee (CNUH-2011-172) according to the ethical guidelines of the 1975 Declaration of Helsinki. All patients provided written informed consent prior to enrollment. In this study, all 8357 patients completed a 2-year clinical follow up by face-to-face interviews, phone calls, or chart review.

### Percutaneous coronary intervention and medical treatments

A diagnostic coronary angiography and PCI were done through either the femoral or the radial artery after an administration of unfractionated heparin (50–100 IU/kg). Patient’s activated clotting time (ACT) was maintained > 250 seconds during the procedure. All patients were given loading doses of 200 to 300mg aspirin and 300 to 600mg clopidogrel before PCI. Revascularization was considered clinically indicated when the patient had typical angina and/or signs of ischemia and ≥ 50% diameter restenosis or ≥ 70% diameter restenosis in a coronary artery by visual estimation. A successful PCI was defined as a residual stenosis <50% and more than Thrombolysis In Myocardial Infarction (TIMI) grade II flow for the infarct related artery (IRA) after the procedure. During in-hospital stay and after discharge, all patients’ medical treatments included aspirin, clopidogrel, beta-blockers (BB), angiotensin converting enzyme inhibitors (ACEI), angiotensin receptor blockers (ARB) and lipid lowering agents.

After discharge, the patients were recommended to stay on the same medications they received during hospitalization. Especially, the total duration of dual antiplatelet therapy (DAPT, the combination of aspirin [100 mg/day] and clopidogrel [75 mg/day]) was recommended for more than 12 months to patients who had undergone PCI. Triple antiplatelet therapy (TAPT) (100mg cilostazol [Pletaal^®^, Otsuka Pharmaceutical Co., Tokyo, Japan]) twice a day added on to DAPT was left to the discretion of the individual operators.

### Study definitions and endpoints

AMI was defined as the presence of clinical symptoms, electrocardiographic changes, or abnormal imaging findings of MI, combined with an increase in the creatine kinase myocardial band fraction above the upper normal limits or an increase in troponin-T/troponin-I to greater than the 99th percentile of the upper normal limit. [15, 16] Smoking status was assessed on the basis of information obtained from hospital medical records at the time of first medical examination and current smoking was defined as a cigarette smoking within 1 year before the index PCI and currently smoke.

The primary endpoint was the occurrence of major adverse cardiac events (MACE) defined as all-cause, recurrent myocardial infarction (re-MI), any coronary revascularization (TLR, target vessel revascularization [TVR], non-TVR) during the 2-year follow-up period. The secondary endpoint was the incidences of definite or probable stent thrombosis (ST). All-cause deaths classified as cardiac (CD) or non-cardiac death. Re-MI was defined as the recurrence of AMI. Any coronary revascularization was defined as a revascularization of the target vessel or non-target vessels. TLR was defined as a revascularization of the target lesion due to restenosis or re-occlusion within the stent or 5mm in and adjacent of the distal or proximal segment. TVR was defined as a revascularization of the target vessel or any segment of the coronary artery containing the target lesion. Non-TVR was defined as a revascularization of any segment of the non-target coronary artery. In addition, Modified American College of Cardiology/American Heart Association criteria were used to classify coronary lesion morphology. [17] Thrombolysis in Myocardial Infarction (TIMI) score was used to determine the degree of coronary flow before and after the procedure. [16]

### Statistical analysis

All statistical analyses were performed using SPSS software, version 20 (SPSS Inc., Chicago, IL, USA). For continuous variables, differences among the three groups were evaluated by analysis of variance or the Jonckheere-Terpstra test, and post-hoc analysis between the two groups was done using the Hochberg test or Dunnett-T3 test; data are expressed as means ± standard deviations. For discrete variables, the differences between the two groups among the three groups were analyzed with the χ^2^ test or Fisher’s exact test, as appropriate; data are expressed as counts and percentages. Multivariable Cox proportional hazards regression, which includes baseline confounding factors, was used to assess independent predictors. We tested meaningful significant (<0.001) available variables that could be of potential relevance: left ventricular ejection fraction (LVEF), ST-segment elevation MI (STEMI), non-ST-segment elevation MI (NSTEMI), creatine kinase myocardial band (CK-MB), clopidogrel, ticagrelor, prasugrel, cilostazole, BB, ACEI, ARB, lipid lowering agent, American College of Cardiology/American Heart Association [ACC/AHA] type B2 and C lesion, 1-vessel disease, 3-vessel disease, stent length, number of stent. Various clinical outcomes were estimated with Kaplan-Meier curve analysis, and differences between groups were compared with the log-rank test. A two-tailed P-value of <0.05 was considered statistically significant.

## Results

### Baseline clinical, angiographic and procedural characteristics

Baseline clinical and laboratory characteristics of this study population are summarized in Table 1. Mean ages of the participants were 57.3±11.5 years and similar among these three groups (p = 0.360). The gender (men) distribution was similar among these three groups (74.9%, p = 0.101). More than 90% of the patients were composed with men. The mean value of LVEF was 52.8±10.9% and relatively well preserved LV systolic function. The numbers of STEMI (63.7%) patients were higher in the ZES group and NSTEMI (42.8%) patients were higher in the BES group compared with other groups. The numbers of hypertension and DM patients were similar among these three groups. In the BES group, even though the prescription rate of clopidogrel (75%) was lower, ticagrelor and prasugrel were more frequently prescribed as discharge medications than the other groups. Angiographic characteristics among these three groups are also summarized in Table 1. LAD was the most common IRA and treated vessel in this study. The incidences of ACC/AHA type B2 lesion, 1-vessel disease, and post-PCI TIMI grade 2 or 3 were higher in the BES group. ACC/AHA type C lesion, 2-vessel disease were higher in the EES group than other groups. The diameter of deployed stent was similar among the three groups. However, the stent length (27.1±11.4mm) and number of stent (1.45±0.76mm) were higher in the EES group.

**Table 1:** Baseline clinical, laboratory, and procedural characteristics.

Variables	ZES (n = 3199)	EES (n = 3987)	ZES and EES (n = 7186)	BES (n = 1171)	P				
					ZES vs. EES	ZES vs. BES	EES vs. BES	ZES/EES vs. BES	ZES vs. EES vs. BES
Age (years)	57.2 ± 11.7	57.6 ± 11.3	57.4 ± 11.5	56.9 ± 11.7	0.101	0.535	0.071	0.176	0.101
Men, n (%)	2978 (93.1)	3751 (94.1)	6729 (93.6)	1117 (95.4)	0.088	0.006	0.088	0.021	0.015
LVEF (%)	52.9 ± 11.0	52.4 ± 10.9	52.6 ± 11.0	53.9 ± 10.3	0.104	0.007	<0.001	<0.001	<0.001
BMI (kg/m^2^)	24.4 ± 3.2	24.3 ±3.2	24.3 ±3.2	24.4 ± 3.2	0.076	0.958	0.223	0.490	0.163
SBP (mmHg)	130.6 ± 27.9	130.3 ± 27.7	130.4 ± 27.8	132.4 ± 27.7	0.647	0.059	0.023	0.025	0.076
DBP (mmHg)	80.3 ± 16.9	80.1 ± 17.0	80.2 ± 17.0	81.5 ± 16.1	0.584	0.037	0.011	0.012	0.045
STEMI, n (%)	2039 (63.7)	2415 (60.6)	4454 (62.0)	670 (57.2)	0.006	<0.001	0.039	0.002	<0.001
NSTEMI, n (%)	1160 (36.3)	1572 (39.4)	2732 (38.0)	501 (42.8)	0.006	<0.001	0.039	0.002	<0.001
Primary PCI, n (%)	1962 (61.3)	2357 (59.1)	4319 (60.1)	661 (56.4)	0.057	0.004	0.103	0.018	0.010
Hypertension, n (%)	1214 (37.9)	1519 (38.1)	2733 (38.0)	413 (35.3)	0.897	0.105	0.079	0.070	0.193
Diabetes mellitus, n (%)	716 (22.4)	903 (22.6)	1619 (22.5)	243 (20.8)	0.788	0.249	0.170	0.175	0.384
Dyslipidemia, n (%)	384 (12.0)	465 (11.7)	849 (11.8)	98 (8.4)	0.656	0.001	0.001	0.001	0.002
Previous MI, n (%)	81 (2.5)	138 (3.5)	219 (3.0)	26 (2.2)	0.023	0.555	0.033	0.120	0.020
Previous PCI, n (%)	137 (4.3)	197 (4.9)	334 (4.6)	41 (3.5)	0.188	0.247	0.039	0.079	0.087
Previous CABG, n (%)	7 (0.2)	10 (0.3)	17 (0.2)	2 (0.2)	0.781	0.756	0.617	0.661	0.873
Previous CVA, n (%)	131 (4.1)	148 (3.7)	279 (3.9)	39 (3.3)	0.404	0.247	0.539	0.360	0.461
Previous HF, n (%)	19 (0.6)	19 (0.5)	38 (0.5)	5 (0.4)	0.495	0.508	0.827	0.652	0.711
CK-MB (mg/dL)	158.4 ± 318.1	141.6 ± 204.8	149.0 ± 261.5	145.6 ± 252.2	0.011	<0.001	0.011	0.002	<0.001
Troponin-I (ng/mL)	59.2 ± 539.7	50.5 ± 116.8	54.3 ± 369.2	52.9 ± 96.3	0.372	0.567	0.523	0.795	0.626
NT-ProBNP (pg/mL)	1123.3 ± 3504.0	1172.5 ± 3510.1	1152.0 ± 3507.3	1044.9 ± 3440.5	0.653	0.621	0.401	0.460	0.693
hs-CRP (mg/dL)	9.8 ± 52.7	7.1 ± 37.7	8.3 ± 45.2	5.5 ± 28.0	0.037	0.003	0.192	0.016	0.019
Serum creatinine (mg/L)	1.1 ± 1.5	1.1 ± 1.0	1.1 ± 1.2	1.0 ± 0.9	0.418	0.026	0.059	0.061	0.119
Total cholesterol (mg/dL)	190.0 ± 44.4	187.1 ± 44.4	188.4 ± 44.4	186.7 ± 44.4	0.007	0.030	0.761	0.220	0.013
Triglyceride (mg/L)	158.4 ± 136.4	151.8 ± 131.1	154.8 ± 133.6	152.1 ± 130.5	0.045	0.171	0.957	0.521	0.106
HDL cholesterol (mg/L)	42.2 ± 13.6	42.3 ± 13.7	42.3 ± 13.7	41.2 ± 10.9	0.797	0.010	0.011	0.002	0.036
LDL cholesterol (mg/L)	120.2 ± 37.8	119.0 ± 38.5	119.5 ± 38.2	119.5 ± 52.8	0.179	0.678	0.748	0.990	0.451
Discharge medications									
Aspirin, n (%)	3017 (94.3)	3798 (95.2)	6811 (94.8)	1126 (96.2)	0.108	0.015	0.152	0.046	0.036
Clopidogrel, n (%)	2837 (88.7)	3265 (81.9)	6102 (84.9)	878 (75.0)	<0.001	<0.001	<0.001	<0.001	<0.001
Ticagrelor, n (%)	162 (5.1)	378 (9.5)	540 (7.5)	121 (10.3)	<0.001	<0.001	0.386	0.001	<0.001
Prasugrel, n (%)	122 (3.8)	230 (5.8)	352 (4.9)	125 (10.7)	<0.001	<0.001	<0.001	0.001	<0.001
Cilostazole, n (%)	629 (19.7)	722 (18.1)	1351 (18.8)	141 (12.0)	0.094	<0.001	<0.001	<0.001	<0.001
ACEI, n (%)	1891 (59.1)	2271 (57.0)	4162 (57.9)	565 (48.2)	0.066	<0.001	<0.001	<0.001	<0.001
ARB, n (%)	622 (19.4)	984 (24.7)	1606 (22.3)	366 (31.3)	<0.001	<0.001	<0.001	<0.001	<0.001
Beta-blocker, n (%)	2512 (78.5)	3320 (83.3)	5832 (81.2)	973 (83.1)	<0.001	0.001	0.885	0.115	<0.001
CCB, n (%)	161 (5.0)	197 (4.9)	358 (5.0)	54 (4.6)	0.859	0.568	0.645	0.587	0.849
Lipid lowering agents	2611 (81.6)	3360 (84.3)	5971 (83.1)	1023 (87.4)	0.003	<0.001	0.009	<0.001	<0.001
Infarct-related artery, n (%)									
LAD, n (%)	1477 (46.2)	1909 (47.8)	3386 (47.1)	586 (50.0)	0.149	0.023	0.193	0.063	0.063
LCx, n (%)	556 (17.4)	658 (16.5)	1214 16.9)	223 (19.0)	0.324	0.203	0.042	0.071	0.121
RCA, n (%)	1111 (34.7)	1329 (33.3)	2440 (34.0)	350 (45.9)	0.214	0.003	0.027	0.006	0.011
Left main, n (%)	52 (1.6)	84 (2.1)	136 (1.9)	9 (0.8)	0.137	0.040	0.002	0.006	0.007
Treated vessel									
LAD, n (%)	1709 (53.4)	2263 (56.8)	3972 (55.3)	662 (56.5)	0.005	0.068	0.891	0.442	0.013
LCx, n (%)	759 (23.7)	992 (24.9)	1751 (24.4)	310 (26.5)	0.257	0.061	0.270	0.121	0.159
RCA, n (%)	1303 (40.7)	1562 (39.2)	2865 (39.9)	415 (35.4)	0.181	0.002	0.021	0.004	0.006
Left main, n (%)	72 (2.3)	113 (2.8)	185 (2.6)	12 (1.0)	0.134	0.009	<0.001	0.001	0.001
ACC/AHA lesion type									
Type B1, n (%)	482 (15.1)	550 (13.8)	1032 (14.4)	177 (15.1)	0.126	0.969	0.254	0.496	0.248
Type B2, n (%)	933 (29.2)	1262 (31.7)	2195 (30.5)	488 (41.7)	0.023	<0.001	<0.001	<0.001	<0.001
Type C, n (%)	1401 (43.8)	1836 (46.0)	3237 (45.0)	442 (37.7)	0.056	<0.001	<0.001	<0.001	<0.001
Extent of coronary artery disease									
1-vessel, n (%)	1669 (52.2)	2022 (50.7)	3691 (51.4)	724 (61.8)	0.219	<0.001	<0.001	<0.001	<0.001
2-vessel, n (%)	928 (29.0)	1218 (30.5)	2146 (29.9)	311 (26.6)	0.156	0.111	0.009	0.021	0.026
≥ 3-vessel, n (%)	589 (18.4)	720 (18.1)	1309 (18.2)	135 (11.5)	0.700	<0.001	<0.001	<0.001	<0.001
Pre-PCI TIMI 0, n (%)	1629 (50.9)	1991 (49.9)	3620 (50.4)	533 (45.5)	0.407	0.002	0.008	0.002	0.006
Post-PCI TIMI 2, n (%)	382 (11.9)	499 (12.5)	881 (12.3)	178 (15.2)	0.461	0.004	0.017	0.005	0.015
Post-PCI TIMI 3, n (%)	634 (19.8)	895 (22.4)	1529 (21.3)	288 (24.6)	0.007	0.001	0.125	0.011	0.001
Stent diameter (mm)	3.20 ± 0.44	3.19 ± 0.44	3.20 ± 0.44	3.19 ± 0.41	0.483	0.716	0.874	0.934	0.775
Stent length (mm)	25.8 ± 9.7	27.1 ± 11.4	26.5 ± 10.7	23.4 ± 8.1	<0.001	<0.001	<0.001	<0.001	<0.001
Number of stent	1.42 ± 0.75	1.45 ± 0.76	1.44 ± 0.76	1.31 ± 0.65	0.063	<0.001	<0.001	<0.001	<0.001

Values are means ± SD or numbers and percentages. The p value for categorical data from chi-square or Fisher’s exact test. ZES, zotarolimus-eluting stents; EES, everolimus-eluting stents; BES, biolimus-eluting stents; LVEF, left ventricular ejection fraction; BMI, body mass index; SBP, systolic blood pressure; DBP, diastolic blood pressure; STEMI, ST-segment elevation myocardial infarction; NSTEMI, non-ST-segment elevation myocardial infarction; MI, myocardial infarction; PCI, percutaneous coronary intervention; CABG, coronary artery bypass graft; CVA, cerebrovascular events; HF, heart failure; CK-MB, creatine kinase myocardial band; NT-ProBNP, N-terminal pro-brain natriuretic peptide; hs-CRP, high-sensitivity C-reactive protein; HDL, high-density lipoprotein; LDL, low-density lipoprotein; ACEI, angiotensin converting enzyme inhibitors; ARB, angiotensin receptor blockers; CCB, calcium channel blockers; ACC/AHA, American College of Cardiology/American Heart Association; TIMI, Thrombolysis In Myocardial Infarction.

### Clinical outcomes

**Fig 2:**
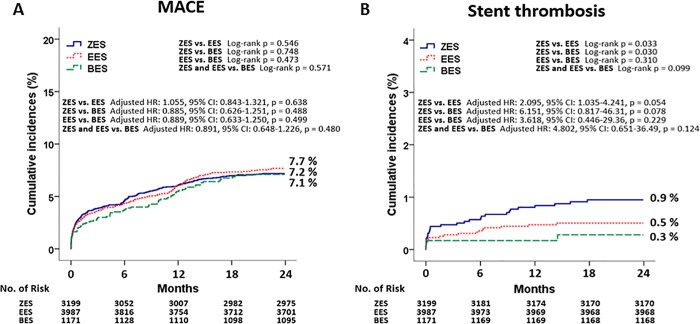
Kaplan-Meier Curved Analysis for MACE (A) and stent thrombosis (B) at 2-year before adjustment. MACE, major adverse cardiac event; ZES, zotarolimus-eluting stent; EES, everolimus-eluting stent; BES, biolimus-eluting stent; HR, hazard ratio; CI, confidence interval.

**Fig 3:**
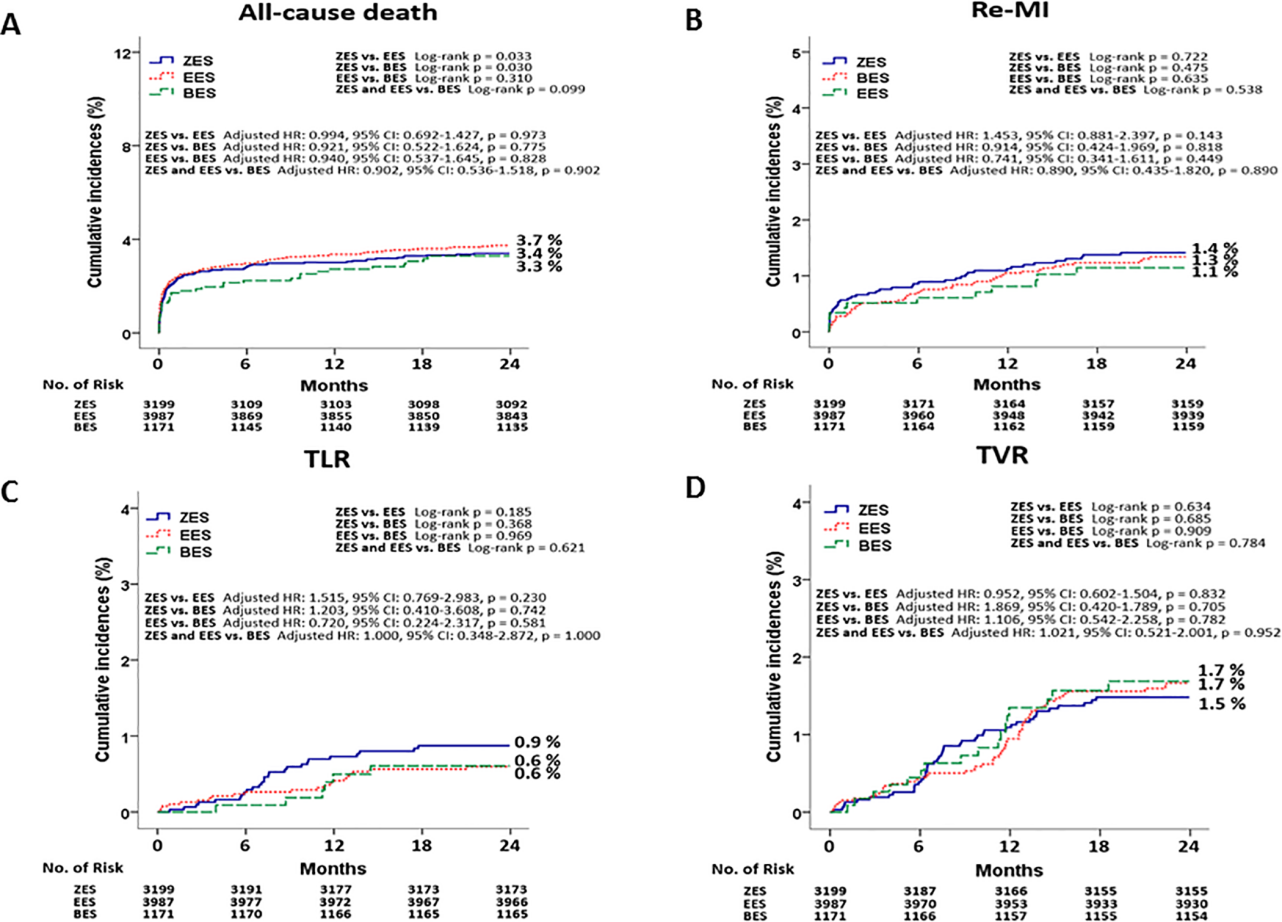
Kaplan-Meier Curved Analysis for all–cause death (A), Re-MI (B), TLR (C), and TVR (D). MI, myocardial infarction; ZES, zotarolimus-eluting stent; EES, everolimus-eluting stent; BES, biolimus-eluting stent; HR, hazard ratio; CI, confidence interval; TLR, target lesion revascularization; TVR, target vessel revascularization.

**Fig 4:**
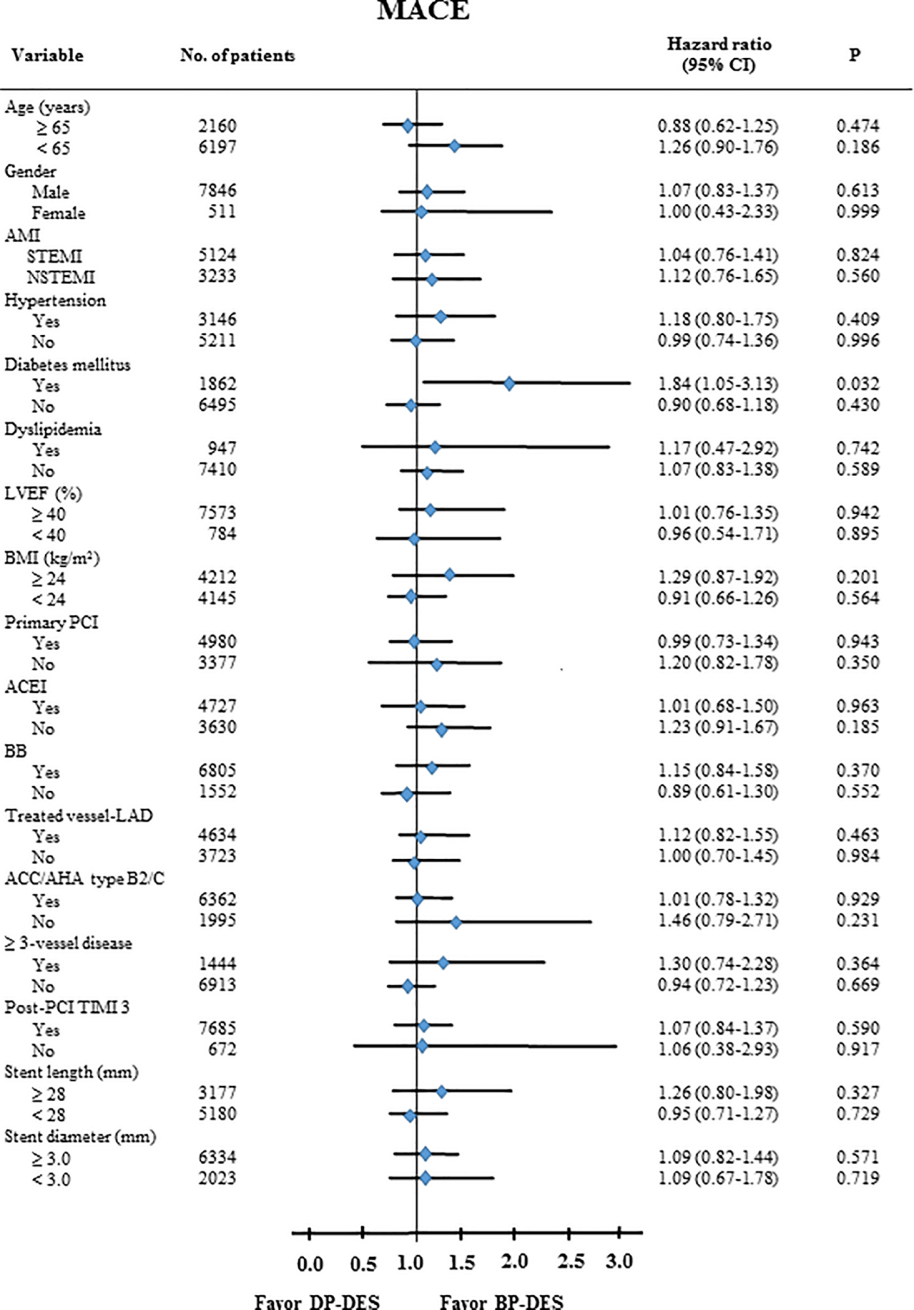
Subgroup analysis for MACE. MACE, major adverse cardiac events; AMI, acute myocardial infarction; STEMI, ST-segment elevation myocardial infarction; NSTEMI, non-ST-segment elevation myocardial infarction; LVEF,: left ventricular ejection fraction; BMI, body-mass index; ACEI, angiotensin converting enzyme inhibitors; BB, beta-blockers; LAD, left anterior descending; ACC/AHA, American College of Cardiology/American Heart Association; PCI, percutaneous coronary intervention; TIMI, Thrombolysis In Myocardial Infarction; DP-DES, durable-polymer drug-eluting stents; BP-DES, biodegradable-polymer DES.

The cumulative incidences of major clinical outcomes at 2 years are listed in Table 2 and Figs 2 and 3. Before adjustment, the cumulative incidence of MACE was not different among these patients (ZES vs. EES = 7.2% vs. 7.7%, Log-rank p = 0.546; ZES vs. BES = 7.2% vs. 7.1%, Log-rank p = 0.748; EES vs. BES = 7.7% vs. 7.1%, Log-rank p = 0.473; ZES/EES vs. BES = 7.4% vs. 7.1%, Log-rank p = 0.571, Fig 2A). In addition, the incidences of all-cause death, cardiac death, TLR, TVR, and non-TVR were not significantly different among these patients. After adjustment, the incidence of MACE were similar among these groups (ZES vs. EES = adjusted hazard ratio [HR], 1.055; 95% confidence interval [CI], 0.843–1.321; p = 0.638; ZES vs. BES = adjusted HR, 0.885; 95% CI, 0.626–1.251; p = 0.488; EES vs. BES = adjusted HR, 0.889; 95% CI, 0.633–1.250; p = 0.499; ZES/EES vs. BES = adjusted HR, 0.891; 95% CI, 0.648–1.226; p = 0.480, Table 3). Other cumulative incidences of all-cause death, CD, MI, any revascularization were similar among these groups before and after adjustment (Table 3). The cumulative incidence of ST was different between ZES vs. EES (ZES vs. EES = 0.9% vs. 0.5%, Log-rank p = 0.033) and ZES vs. BES (ZES vs. BES = 0.9% vs. 0.3%, Log-rank p = 0.030) before adjustment in Table 2 and Fig 2. However, after adjustment, the incidence of ST was not significantly different between ZES vs. EES (adjusted HR, 2.095; 95% CI, 1.035–4.241; p = 0.054) and ZES vs. BES (adjusted HR, 6.151; 95% CI, 0.817–46.31; p = 0.078) (Table 3). Moreover, the comparison between EES vs BES (adjusted HR, 3.618; 95% CI, 0.446–29.36; p = 0.229) and ZES/EES vs. BES (adjusted HR, 4.802; 95% CI, 0.651–36.49; p = 0.124) also showed similar results. Table 4 shows the independent predictors for MACE and ST at 2 years. Age ≥65 years, LVEF <40%, aspirin, stent diameter were significant independent predictors for MACE. By contrast, hypertension and stent diameter was significant independent predictor for ST in this study. Fig 4 shows subgroup analysis for MACE. All variables except for DM shows comparable results for MACE between DP-DES and BP-DES. In case of DM, BP-DES was preferred results for MACE (HR, 1.84; 95% CI, 1.05–3.13; p = 0.032) compared with DP-DES.

**Table 2:** Cumulative clinical events at 2 years before adjustment.

Variables	ZES (n = 3199)	EES (n = 3987)	ZES and EES (n = 7186)	BES (n = 1171)	P			
					ZES vs. EES	ZES vs. BES	EES vs. BES	ZES/EES vs. BES
Primary outcome								
MACE, n (%)	224 (7.0)	286 (7.2)	510 (7.1)	76 (6.5)	0.779	0.553	0.421	0.451
All-cause death, n (%)	107 (3.3)	144 (3.6)	251 (3.5)	36 (3.1)	0.540	0.656	0.378	0.466
Cardiac death, n (%)	93 (2.9)	113 (2.8)	206 (2.9)	26 (2.2)	0.854	0.217	0.254	0.212
Re-MI, n (%)	43 (1.3)	48 (1.2)	91 (1.3)	12 (1.0)	0.597	0.402	0.615	0.487
Any revascularization, n (%)	82 (2.6)	118 (3.0)	200 (2.8)	31 (2.6)	0.310	0.877	0.575	0.793
TLR, n (%)	26 (0.8)	21 (0.5)	47 (0.7)	6 (0.5)	0.135	0.302	0.952	0.571
TVR, n (%)	44 (1.4)	57 (1.4)	101 (1.4)	17 (1.5)	0.846	0.849	0.955	0.901
Non-TVR, n (%)	39 (1.2)	62 (1.6)	101 (1.4)	16 (1.4)	0.229	0.699	0.642	0.916
Secondary outcome								
Stent thrombosis (probable or definite), n (%)	29 (0.9)	19 (0.5)	48 (0.7)	3 (0.3)	0.029	0.026	0.309	0.093

Values are means ± SD or numbers and percentages. The p value for categorical data from chi-square or Fisher’s exact test. ZES, zotarolimus-eluting stents; EES, everolimus-eluting stents; BES, biolimus-eluting stents; MACE major adverse cardiac events; Re-MI, recurrent myocardial infarction; TLR target lesion revascularization, TVR target vessel revascularization, Non-TVR non-target vessel revascularization

**Table 3:** Hazard ratio for 2-year major clinical outcomes according to the type of stent by Cox-proportional hazard ratio analysis.

**HR (95% confidence interval), P**				
	**ZES vs. EES**	**ZES vs. BES**	**EES vs. BES**	**ZES/EES vs. BES**
**Primary endpoint**				
MACE				
Unadjusted	0.948 (0.795–1.129), 0.546	1.044 (0.804–1.354), 0.748	1.097 (0.852–1.413), 0.473	1.072 (0.843–1.364), 0.571
*Adjusted	1.055 (0.843–1.321), 0.638	0.885 (0.626–1.251), 0.488	0.889 (0.633–1.250), 0.499	0.891 (0.648–1.226), 0.480
All-cause death				
Unadjusted	0.909 (0.708–1.167), 0.455	1.065 (0.730–1.554), 0.742	1.173 (0.814–1.691), 0.391	1.126 (0.794–1.596), 0.507
*Adjusted	0.994 (0.692–1.427), 0.973	0.921 (0.522–1.624), 0.775	0.940 (0.537–1.645), 0.828	0.902 (0.536–1.518), 0.902
Cardiac death				
Unadjusted	1.013 (0.770–1.333), 0.928	1.291 (0.835–1.994), 0.250	1.279 (0.835–1.958), 0.258	1.285 (0.855–1.933), 0.228
*Adjusted	0.995 (0.658–1.503), 0.981	0.920 (0.472–1.793), 0.808	1.009 (0.522–1.948), 0.980	0.965 (0.522–1.787), 0.910
Re-MI				
Unadjusted	1.078 (0.714–1.626), 0.722	1.262 (0.666–2.394), 0.476	1.166 (0.619–2.194), 0.635	1.208 (0.662–2.205), 0.539
Adjusted	1.453 (0.881–2.397), 0.143	0.914 (0.424–1.969), 0.818	0.741 (0.341–1.611), 0.449	0.890 (0.435–1.820), 0.890
Any Revascularization				
Unadjusted	0.821 (0.620–1.089), 0.171	0.918 (0.607–1.388), 0.684	1.104 (0.743–1.639), 0.625	1.016 (0.696–1.483), 0.934
*Adjusted	0.840 (0.605–1.166), 0.298	0.842 (0.500–1.419), 0.519	1.115 (0.678–1.833), 0.669	1.001 (0.623–1.610), 0.996
TLR				
Unadjusted	1.472 (0.828–2.615), 0.188	1.500 (0.617–3.644), 0.371	1.018 (0.411–2.522), 0.969	1.239 (0.530–2.898), 0.621
*Adjusted	1.515 (0.769–2.983), 0.230	1.203 (0.401–3.608), 0.742	0.720 (0.224–2.317), 0.581	1.000 (0.348–2.872), 1.000
TVR				
Unadjusted	0.909 (0.613–1.347), 0.635	0.891 (0.509–1.559), 0.686	0.969 (0.564–1.665), 0.909	0.931 (0.557–1.556), 0.784
*Adjusted	0.952 (0.602–1.504), 0.832	0.869 (0.420–1.798), 0.705	1.106 (0.542–2.258), 0.782	1.021 (0.521–2.001), 0.952
Non-TVR				
Unadjusted	0.746 (0.500–1.114), 0.152	0.851 (0.476–1.524), 0.588	1.127 (0.650–1.952), 0.670	0.999 (0.589–1.692), 0.996
*Adjusted	0.734 (0.460–1.171), 0.194	0.679 (0.336–1.372), 0.281	0.960 (0.502–1.835), 0.902	0.837 (0.450–1.556), 0.574
**Secondary endpoint**				
Stent thrombosis				
Unadjusted	1.856 (1.041–3.310), 0.036	3.436 (1.047–11.28), 0.042	1.860 (0.550–6.285), 0.318	2.576 (0.802–8.271), 0.112
*Adjusted	2.095 (1.035–4.241), 0.054	6.151 (0.817–46.31), 0.078	3.618 (0.446–29.36), 0.229	4.802 (0.651–36.49), 0.124

*Adjusted model was included LVEF, STEMI, NSTEMI, CK-MB, clopidogrel, ticagrelor, prasugrel, cilostazole, ACEI, ARB, BB, lipid lowering agent, ACC/AHA type B2 and C lesion, 1-vessel disease, 3-vessel disease, stent length, number of stent

LVEF, left ventricular ejection fraction; STEMI, ST-segment elevation myocardial infarction, NSTEMI, non-ST-segment elevation myocardial infarction; CK-MB, creatine kinase myocardial band; ACEI, angiotensin converting enzyme inhibitor; ARB, angiotensin receptor blocker; BB, beta blocker, ACC/AHA, American College of Cardiology/American Heart Association. (We do not want color text)

**Table 4:** Independent predictors for MACE and stent thrombosis at 2 years.

	MACE				Stent thrombosis			
	Unadjusted		Adjusted		Unadjusted		Adjusted	
Variables	HR (95% CI)	P	HR (95% CI)	P	HR (95% CI)	P	HR (95% CI)	P
Age≥65 years	2.224 (1.888–2.620)	<0.001	1.633 (1.267–2.103)	<0.001	0.805 (0.413–1.569)	0.524	0.726 (0.286–1.843)	0.500
Gender (men)	0.598 (0.455–0.785)	<0.001	0.822 (0.555–1.217)	0.327	0.760 (0.274–2.111)	0.760	0.385 (0.125–1.189)	0.097
STEMI	1.059 (0.896–1.525)	0.503	1.070 (0.530–2.162)	0.850	1.374 (0.761–2.483)	0.292	3.426 (0.704–16.67)	0.127
LVEF<40%	2.803 (2.273–3.458)	<0.001	1.554 (1.136–2.125)	0.006	0.831 (0.299–2.312)	0.723	0.392 (0.088–1.743)	0.219
BMI≥24kg/m^2^	0.727 (0.613–0.862)	<0.001	1.012 (0.801–1.277)	0.923	0.849 (0.485–1.486)	0.567	0.888 (0.444–1.778)	0.738
Primary PCI	1.044 (0.885–1.233)	0.607	0.949 (0.474–1.899)	0.883	1.238 (0.697–2.199)	0.466	0.392 (0.087–1.758)	0.221
Systolic blood pressure	0.993 (0.989–0.996)	<0.001	0.999 (0.992–1.007)	0.855	1.001 (0.991–1.011)	0.902	0.992 (0.969–1.016)	0.527
Diastolic blood pressure	0.987 (0.982–0.992)	<0.001	1.001 (0.989–1.013)	0.893	1.001 (0.985–1.018)	0.858	1.022 (0.985–1.061)	0.254
Hypertension	1.231 (1.045–1.451)	0.013	1.002 (0.792–1.269)	0.985	0.569 (0.303–1.067)	0.079	0.373 (0.156–0.895)	0.027
Diabetes mellitus	1.614 (1.172–2.223)	0.003	0.992 (0.744–1.322)	0.954	1.092 (0.572–2.086)	0.789	1.464 (0.595–3.603)	0.407
Dyslipidemia	0.850 (0.648–1.115)	0.240	0.993 (0.672–1.469)	0.974	1.036 (0.442–2.429)	0.935	1.515 (0.607–3.782)	0.373
Aspirin	0.177 (0.145–0.217)	<0.001	0.446 (0.245–0.812)	0.008	0.725 (0.226–2.328)	0.589	0.965 (0.152–6.141)	0.970
Clopidogrel	0.918 (0.733–1.148)	0.453	0.883 (0.418–1.864)	0.743	1.613 (0.640–4.064)	0.310	0.920 (0.116–7.275)	0.937
Ticagrelor	0.660 (0.441–0.986)	0.042	0.369 (0.108–1.262)	0.112	0.565 (0.137–2.327)	0.429	0.835 (0.321–2.175)	0.713
Prasugrel	0.618 (0.396–0.965)	0.034	0.780 (0.303–2.012)	0.608	0.346 (0.048–2.502)	0.293	0.430 (0.026–7.205)	0.557
Cilostazole	0.836 (0.671–1.042)	0.111	0.889 (0.641–1.233)	0.482	1.350 (0.707–2.579)	0.363	0.545 (0.204–1.459)	0.227
ACEI	0.532 (0.451–0.626)	<0.001	0.877 (0.621–1.239)	0.457	0.653 (0.377–1.131)	0.129	0.739 (0.310–1.759)	0.494
ARB	0.831 (0.679–1.016)	0.071	1.162 (0.796–1.694)	0.437	0.697 (0.339–1.432)	0.326	0.622 (0.205–1.887)	0.401
Beta blocker	0.351 (0.297–0.415)	<0.001	0.758 (0.541–1.063)	0.108	0.565 (0.306–1.046)	0.069	0.738 (0.297–1.829)	0.511
Lipid lowering agent	0.390 (0.328–0.464)	<0.001	0.836 (0.585–1.194)	0.325	1.020 (0.480–2.170)	0.959	0.860 (0.331–2.236)	0.757
Treated vessel-LAD	1.124 (0.954–1.324)	0.163	1.310 (0.837–2.052	0.238	0.987 (0.569–1.713)	0.963	0.764 (0.203–2.875)	0.691
Treated vessel-RCA	1.106 (0.939–1.303)	0.229	0.789 (0.468–1.330)	0.373	1.000 (0.570–1.754)	0.999	0.860 (0.331–2.236)	0.757
Treated vessel-LM	2.879 (2.055–4.033)	<0.001	1.135 (0.402–3.209)	0.811	2.699 (0.841–8.665)	0.095	0.527 (0.104–2.677)	0.440
ACC/AHA type B2 lesion	1.075 (0.905–1.276)	0.411	1.140 (0.831–1.599)	0.447	0.888 (0.486–1.623)	0.700	1.291 (0.503–3.317)	0.596
Pre-PCI TIMI 0	1.064 (0.905–1.251)	0.453	0.981 (0.746–1.291)	0.893	1.452 (0.832–2.537)	0.190	1.333 (0.629–2.824)	0.453
Post-PCI TIMI 3	0.948 (0.711–1.263)	0.714	1.800 (0.855–3.788)	0.122	1.433 (0.446–4.601)	0.546	0.513 (0.139–1.887)	0.315
Stent diameter	0.699 (0.569–0.834)	<0.001	0.620 (0.451–0.853)	0.003	0.419 (0.211–0.834)	0.013	0.431 (0.186–1.000)	0.050
Stent length	1.013 (1.006–1.020)	<0.001	1.009 (0.998–1.020)	0.118	1.009 (0.985–1.034)	0.472	0.982 (0.943–1.023)	0.379

MACE, major adverse cardiac events; HR, hazard ratio; STEMI, ST-segment elevation myocardial infarction myocardial infarction; LVEF, left ventricular ejection fraction; BMI, body mass index; PCI, percutaneous coronary intervention; ACEI, angiotensin converting enzyme inhibitor; ARB, angiotensin receptor blocker; LAD, left anterior coronary artery; RCA, right coronary artery; LM, left main; ACC/AHA, American College of Cardiology/American Heart Association; TIMI, thrombolysis in myocardial infarction.

## Discussion

In this study, we investigated the impact of current smoking on 2-year clinical outcomes between DP-coated DES (ZES and EES) and BP-coated BES in AMI patients after successful PCI. The main findings of this study are as follows; 1) The MACE and ST rates were similar between DB-DES and BP-DES in patients with AMI with current smoking after successful PCI during 2-year follow-up period and 2) the 2-year adjusted HR for all-cause death, CD, re-MI, TLR, TVR, and non-TVR were not significantly different.

Even though, there are some debates, Huang et al. [18] demonstrated that persistent smoking increased neointimal hyperplasia area (1.04±0.72 mm^2^ vs 0.96±0.68 mm^2^; p = 0.04) and malapposed struts (3.2% vs 1.6%; p = 0.004) compared with non-smoker. In addition, persistent smoking cause a high incidence of uncovered struts if the duration of smoking was more than 1-year. However, these results were obtained from the patients who underwent 1G-DES (sirolimus-eluting stent [SES]) were deployed. Athough, the majority of 2G-DES showed non-inferior clinical outcomes compared with 1G-DES, [19, 20] these durable-polymer based stents have been associated with persistent local inflammatory and toxic reactions, delayed healing, hypersensitivity reactions, endothelial dysfunction, and neo-atherosclerosis. [21, 22] Taken together, the BP-DES is became interested recently. The polymer of BP-BES is consisted with polylactic acid and that is fully degraded into carbon dioxide and water within 6 months. [23] In spite of this peculiar advantage of BP, the long-term clinical outcome are debatable compared with DP. [24, 25] This study also showed that the incidences of MACE (adjusted HR, 0.891; 95% CI, 0.648–1.226; p = 0.480) and ST (adjusted HR, 4.802; 95% CI, 0.651–36.49; p = 0.124) of BP-DES were similar compared with DP-DES regardless of current smoking. According this results we can assume that the hazardous effect of current smoking was not influenced by the presence or absence of polymer of new-generation DES in this study. As mentioned, nicotine may play an important role in atherogenesis and involved in enhanced endothelial cell proliferation and migration, and accelerate intimal hyperplasia in vitro and animal study. However, the operative mechanisms at the level of endothelium are not clearly understood. [7] Other possible mechanisms are related with its hemodynamic influences including increase in blood pressure and heart rate, and decreased exercise tolerance and pro-coagulant effect. [26] Current smoking also increase inflammation and oxidative damage to the vascular endothelium and impair coronary circulatory function. [27] The relationship between stent strut thickness and platform design and long-term safety and efficacy of DES was not well defined. One ex-vivo model showed polymer coatings and drugs do not increase the risk of acute ST, but rather serve as corrosive barriers and decrease the risk. [28] Another a porcine model study showed that similar inflammatory histomorphometric reaction between DP-DES and BP-DES. [29] Some other studies [30, 31] suggested that the concept that DP are key in very late ST may be challengeable and may not have clinical significance. In this study, the occurrence of ST was different between ZES vs. EES and ZES vs. BES before adjustment. However, after adjustment, the incidence of ST was not significantly different between ZES vs. EES (adjusted HR, 2.095; 95% CI, 1.035–4.241; p = 0.054) and ZES vs. BES (adjusted HR, 6.151; 95% CI, 0.817–46.31; p = 0.078), EES vs BES (adjusted HR, 3.618; 95% CI, 0.446–29.36; p = 0.229) and ZES/EES vs. BES (adjusted HR, 4.802; 95% CI, 0.651–36.49; p = 0.124). These results also cautiously suggest that current smoking’s effect on total occurrence of ST according to type of polymer (DB vs. BP) in AMI patients were not significantly different. However, even though these comparisons between DP-DES and BP-DES showed statistically insignificant due to wide confidence interval, their absolute value of adjusted HR could be numerically meaningful and significant. Therefore, these results of this study need to reevaluated by large-scaled randomized controlled studies in the future. As a result, although, this study demonstrated comparable major clinical outcomes between DP-DES and BP-DES in AMI patients with current smoking during 2-year follow-up period after successful PCI, we think that smoking is modifiable risk factor for cardiovascular disease and cessation of smoking is much more important than the presence or absence of polymer during PCI in the new-generation DES era.

Critchley et al [32] reported that smoking cessation decreased about a 36% crude relative risk (RR) of mortality for patients with coronary heart disease compared with continued smoking (RR, 0.64; 95% CI, 0.58–0.71). This beneficial effect of smoking cessation can be achieved by vascular healing after stent deployment through decrease the progression of neointimal hyperplasia and decrease the incidence of stent malapposition. [18] However, even if the patients had stopped smoking during hospitalization, the complete cessation of cigarette smoking after PCI is very difficult challenge in clinical practice and which may cause severe adverse clinical events. Even though there were no established data, the smoking status of the AMI patients may be more likely to change longitudinally than elective PCI. The rates of successful smoking cessation after PCI was approximately 40–80%. [33, 34]. According these reports we can assume that about 20–60% of enrolled patients of this study may be still current smoker after index PCI during 2-year follow-up period at that time. Therefore, in this study, even though smoking status of the study population was assessed at the time of PCI, the results of this study may give some meaningful message in interventional cardiologist during PCI especially, in AMI patients with current smoking.

In this study, there were several limitations. First, the present study was non-randomized study, similar to every “real-world” registry; there may be some under-reporting and/or missed data. Second, smoking status was very important in this study. However, smoking status of the study population was assessed at the time of PCI, we did not know the precise smoking status during follow-up period. This may can affect the results of this study. In addition, the data concerning the quantity of smoking at the time of PCI was not perfectly evaluated. Third, because in case of prescribed medications which based on the medications at discharge and this registry data did not include the detailed full data concerning the prescription doses, long-term adherence, discontinuation, drug-related adverse events, this factor may act as an important bias in this study. Fourth, although we did multivariable Cox-proportional regression analysis to overcome the limitation of this retrospective study, the characteristics of this retrospective registry might have influence the results of this study. Fifth, because the choice of ZES, EES, or BES was depended on the discretion of the physician, this may be important bias in this study.

## Conclusions

In conclusion, the AMI patients with current smoking underwent successful PCI with DB-DES or BP-DES showed comparable safety and efficacy during 2-year follow-up periods. Therefore, DP-DES or BP-DES are equally acceptable in AMI patients with current smoking undergoing PCI. However, this result maybe more precisely be defined by other well-designed prospective studies in the future.
